# Anticancer Potential of Thiocolchicoside: An In-Vitro MTT Assay Study

**DOI:** 10.7759/cureus.109395

**Published:** 2026-05-21

**Authors:** Akash V Devi, Chitra Khanwelkar

**Affiliations:** 1 Pharmacology, Krishna Vishwa Vidyapeeth Deemed to be University, Karad, IND

**Keywords:** cell lines, drugs repurposing, mtt assay, oncology, pharmacology and therapeutics

## Abstract

Thiocolchicoside (THC), a semi-synthetic derivative of colchicoside, is utilized clinically as a muscle relaxant. Nonetheless, its structural resemblance to colchicine indicates possible anticancer attributes. This study sought to assess the cytotoxic effects of THC on human mammary gland breast adenocarcinoma (MCF-7) and human gastric adenocarcinoma (AGS) cell lines utilizing the 3-(4,5-dimethylthiazol-2-yl)-2,5-diphenyltetrazolium bromide (MTT) assay, in comparison to VC. Cells were exposed to incremental dosages of THC 20-100 µg/ml and VC 20-100 µg/ml for 24 to 48 hours, and cell viability was evaluated. THC showed dose‑dependent cytotoxicity, reducing cell viability at higher concentrations, though less potent than VC. These results suggest that THC may have anticancer properties, necessitating more mechanistic and in vivo investigations. The aim was to study the anticancer effects of THC in various cell lines in comparison with the standard drug VC. The MCF-7 and AGS cell lines, which were chosen as exemplary models for the investigation, were procured from the National Centre of Cell Sciences (NCCS), Pune, India. Dimethyl Sulfoxide (DMSO), 200 µl, which was used as a control in this study, was procured from Thermo Fisher, Pune, India. THC was used as a test drug and was procured from Dr. Reddy's Laboratory Limited, Hyderabad, India, and VC, which was used as a reference drug, was procured from Yarrow Chem Products, Mumbai, India. The MTT reagent used in this study to monitor cell viability was procured from Thermo Fisher Scientific, Pune, India. Cell lines were treated with different concentrations of THC and VC ranging from 20 to 100 µg/ml for 24-48 hours, and absorbance was measured at 570 nm. After treatment, their cell viability was measured. VC exhibited markedly superior inhibitory action compared to THC in MCF-7 cells (p < 0.05); however, in AGS cells, the difference was not statistically significant (p > 0.05), suggesting comparable effects under the evaluated conditions. This shows THC has moderate cytotoxic activity. THC showed a dose-dependent decrease in cell viability and can be selected as a repurposing agent.

## Introduction

Cancer continues to be one of the most prevalent causes of mortality worldwide, resulting in millions of fatalities each year [1,2]. A promising strategy for identifying new therapeutic applications for existing pharmaceuticals has emerged in recent years: drug repurposing [3]. This approach reduces the time, cost, and risk associated with de novo drug development. Thiocolchicoside (THC) is a semi-synthetic derivative of colchicoside that was initially isolated from the seeds of Gloriosa superba and is a member of the Liliaceae family [4]. Due to its central neuromuscular activity, THC has been extensively employed as a muscle relaxant in clinical settings, offering respite from rheumatologic diseases, inflammatory conditions, and musculoskeletal disorders. It is an appealing candidate for repurposing due to its well-established pharmacological profile. THC is structurally similar to colchicine, a well-known microtubule inhibitor that has exhibited potent anticancer activity [5,6]. Colchicine and its derivatives induce apoptosis and mitotic arrest in cancer cells by binding to tubulin, disrupting microtubule dynamics, and thereby exerting cytotoxic effects [7-9]. This structural similarity implies that THC may also have anticancer properties. Preliminary research has suggested that THC has the potential to induce apoptosis by upregulating tumor suppressor proteins like p53 and by inhibiting the NF-κB signaling pathway, which is frequently linked to the survival and proliferation of cancer cells [10-13]. Although these promising discoveries have been made, there is still a lack of comprehensive evaluation of their anticancer efficacy. As a result, the current study was devised to examine the cytotoxic effects of THC on human mammary gland breast adenocarcinoma (MCF‑7) and human gastric adenocarcinoma (AGS) cell lines, with vincristine (VC) serving as the reference standard. The objective of this study is to substantiate the potential repurposing of THC as an anticancer agent with additional evidence.

Primary objective

To assess the cytotoxic effects of THC on Human Mammary Gland Breast Adenocarcinoma (MCF‑7) and Human Gastric Adenocarcinoma (AGS) cell lines using the MTT assay, in comparison to vincristine (VC) as a reference standard.

Secondary objective 

To evaluate the potential of THC as a repurposed anticancer agent by comparing its activity across various cell lines and evaluating its dose-dependent cytotoxicity.

## Materials and methods

Each experimental condition was evaluated in triplicate wells (n = 3) to ensure repeatability. Cells were randomly assigned to wells to reduce bias, and untreated controls were included concurrently. The sample size was selected according to conventional protocols for in vitro cytotoxicity experiments, ensuring enough statistical power for IC₅₀ determination and paired t-test analysis. The human mammary gland breast adenocarcinoma (MCF-7) and human gastric adenocarcinoma (AGS) cell lines were chosen as exemplary models for the investigation [14-15]. THC (purity ≥98%) and VC were procured from accredited vendors.

The 3‑(4,5‑dimethylthiazol‑2‑yl)‑2,5‑diphenyltetrazolium bromide (MTT) assay, an established colorimetric method for monitoring cell viability, was used to evaluate cells for cytotoxicity [16,17]. Cells were placed in 96-well plates and given different amounts of THC (20-100 µg/ml) and VC (20-100 µg/ml) for 24-48 hours. After incubation, MTT reagent was added, and the absorbance was measured at 570 nm. The vitality of cells was quantified as a percentage in comparison to untreated controls. We used dose-dependent response data and regression analysis to find IC₅₀ values and paired t-test statistical significance, with p < 0.05 being significant. Regression equations and R² values were shown on the plots, and IC₅₀ was determined as the concentration yielding 50% inhibition. Table 1 summarizes varying dosages of THC and VC, which were used in this study.

**Table 1:** Drug concentrations of THC and VC were tested against MCF 7 and AGS cell lines. MCF-7: Human mammary gland breast adenocarcinoma cell lines, AGS-: Human gastric adenocarcinoma cell lines, µg/mL: Microgram per milliliter, THC: Thiocolchicoside, VC: Vincristine

Concentration (µg/mL)	
THC	VC
20	20
40	40
60	60
80	80
100	100

Equipment

MCF-7 and AGS were obtained from the National Center for Cell Sciences (NCCS), Pune, India. Cell culture and test setup were done on 96-well plates. Carbon dioxide (CO₂) incubator BB150, which was used to keep the cells at a constant temperature and humidity. A biosafety cabinet was used to handle biological samples in a sterile way. Enzyme-Linked Immunosorbent Assay (ELISA) plate reader (Benesphera E21) to measure absorbance in microplate-based tests.

Ethical aspects

All experiments were conducted using authenticated cell lines obtained from recognized repositories. There were no human volunteers or animal subjects directly involved in this investigation. Institutional biosafety regulations were rigorously adhered to, encompassing aseptic procedures in certified biosafety cabinets and routine contamination testing. The Institutional Ethics Committee (IEC) looked over the study and decided that it didn't need human or animal ethics approval because it followed the rules. Data integrity and transparency were upheld consistently, assuring adherence to international ethical norms for cell line research. Clearance to conduct the research was obtained from the IEC with protocol approval number 616/2023-2024.

Chemical and drugs

The MTT reagent was procured from Thermo Fisher Scientific, Pune, India. THC (active pharmaceutical ingredient) was procured from Dr. Reddy's Laboratory Limited, Hyderabad, India. VC (Active Pharmaceutical Ingredient) was procured from Yarrow Chem Products, Mumbai, India. Minimal Essential Medium (MEM) with high glucose was procured from Thermo Fisher Scientific, Pune, India, with catalogue number 11965-092. Fetal Bovine Serum (FBS) was procured from Gibco, Invitrogen India, with catalogue number 10270106. Dimethyl Sulfoxide (DMSO) was procured from Thermo Fisher Scientific, Pune, India. Penicillin-Streptomycin (10,000 U/mL) was procured from Thermo Fisher Scientific, Pune, India. Trypsin-EDTA (0.25%) and phenol red (100 ml) were procured from Thermo Fisher Scientific, Pune, India.

Experimental model

MTT Assay

**Figure 1:**
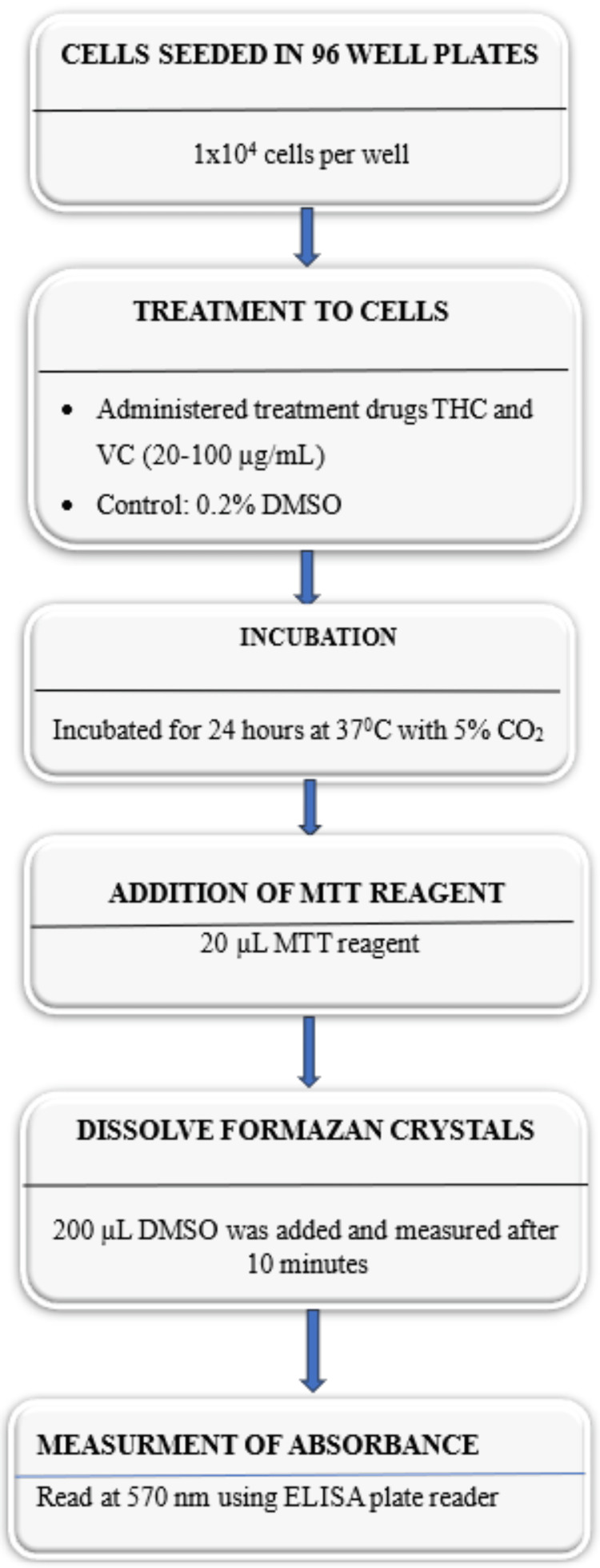
Illustration of methodology of MTT assay. THC: Thiocolchicoside, VC: Vincristine, DMSO: Dimethyl-sulfoxide, MCF-7: Human Breast Adenocarcinoma Cell Lines, AGS-: Human Gastric Adenocarcinoma Cell Lines, µg/mL: Microgram per milliliter, nm: Nano-meter

Cells were kept in culture medium at a concentration of 1 × 10⁴ cells/ml for 24 hours at 37°C with 5% CO₂. For seeding, 70 μl of a solution with 10⁴ cells/well was added to 100 μl of culture media, and then 100 μl of test samples (20-100 μg/ml) was applied to tissue culture grade 96-well plates. DMSO (0.2%) in PBS was used as a control. All samples were processed in triplicate to assess cell viability. A CO₂ incubator (Thermo Scientific BB150) was used to keep the cell cultures at 37°C with 5% CO₂ for 24 hours. After the incubation, the medium was taken out, and 20 μl of MTT reagent (5 mg/ml in PBS) was added. The mixture was then put back in the CO₂ incubator for 4 hours at 37°C. We looked at wells under a microscope to see if formazan crystals were forming. This happened when living cells turned yellow MTT into dark-colored formazan. After the medium was completely removed, 200 μl of DMSO was added, left for 10 minutes, and then incubated at 37°C while wrapped in aluminum foil. Lastly, we used an ELISA microplate reader to measure the absorbance at 570 nm of three samples [16,17]. Figure 1 illustrates the methodology of the MTT assay procedure.

Statistical analysis

Statistical analysis was done using GraphPad InStat version 3.06 (GraphPad Software Inc., San Diego, California, USA). A paired t-test was used to determine the statistical significance of the percentage inhibition induced by vincristine and thiocolchicoside at equal doses in various cancer cell lines. The tested MCF-7 cell line, it showed a statistically significant difference between the two drugs (t = 4.685, p = 0.0094; p < 0.05). These results showed that VC had much stronger inhibitory activity than THC under the tested conditions. The tested AGS cell line showed a t-value of 2.518 and a p = 0.0655 (p > 0.05). The differences between VC and THC were comparable.

## Results

Thiocolchicoside (THC) reduced cell viability in a concentration-dependent manner in both AGS and MCF-7 cell lines, as demonstrated by the MTT assay. VC, the standard drug, consistently exhibited reduced IC₅₀ values, underscoring its increased potency. In particular, THC demonstrated IC₅₀ values of 43.82 µg/mL in MCF‑7 and 47.38 µg/mL in AGS, while VC exhibited values of 29.71 µg/mL and 37.82 µg/mL, respectively. These results corroborated that THC exhibited measurable antiproliferative activity; however, VC is more potent, as it necessitates lower concentrations to achieve 50% inhibition (Table 2).

**Table 2:** Comparison of IC50 values for MCF-7 and AGS cell lines against VC and THC. VC: Vincristine, THC: Thiocolchicoside, IC50: Half Maximal Inhibitory Concentration, µg/mL: Microgram per milliliter, MCF-7: Human mammary gland breast adenocarcinoma cell line, AGS: Human gastric adenocarcinoma cell line

Cell Line	Drugs	IC₅₀ (µg/mL)
MCF-7	VC	29.71
MCF-7	THC	43.82
AGS	VC	37.82
AGS	THC	47.38

**Figure 2:**
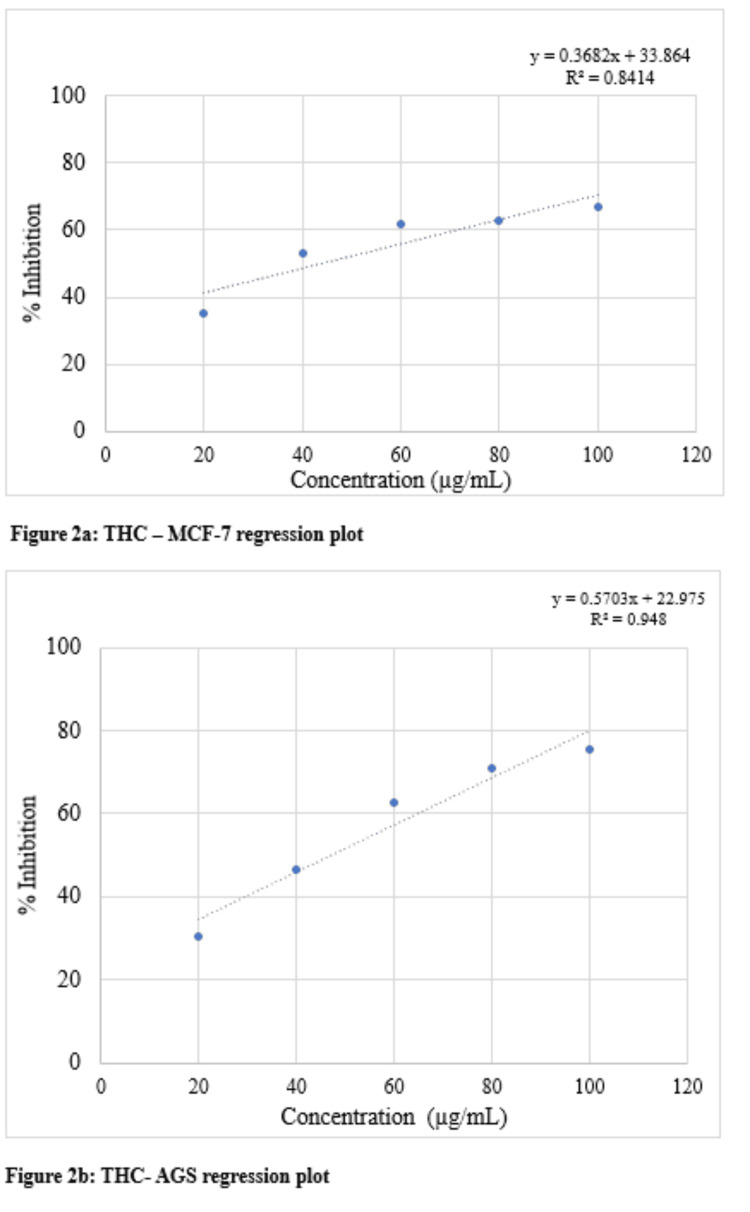
a) Linear regression plot for THC-MCF-7 b) Linear regression plot for THC-AGS. THC: Thiocolchicoside, MCF-7: Human mammary gland breast adenocarcinoma cell line, AGS: Human gastric adenocarcinoma cell line, µg/mL: Microgram per milliliter, %: Percentage

Figure 2 depicts the dose-dependent inhibitory impact of THC on human cancer cell lines. Linear regression analysis demonstrated a robust connection between elevated concentrations of THC and percentage inhibition in both MCF-7 (breast cancer) and AGS (gastric adenocarcinoma) cells. For MCF-7, the regression equation was y = 0.3682x + 33.864, with R² = 0.8414, but AGS cells exhibited a superior fit with y = 0.5703x + 22.975 and R² = 0.948. The data demonstrate that THC has a concentration-dependent cytotoxic effect, with AGS cells exhibiting greater sensitivity.

**Figure 3:**
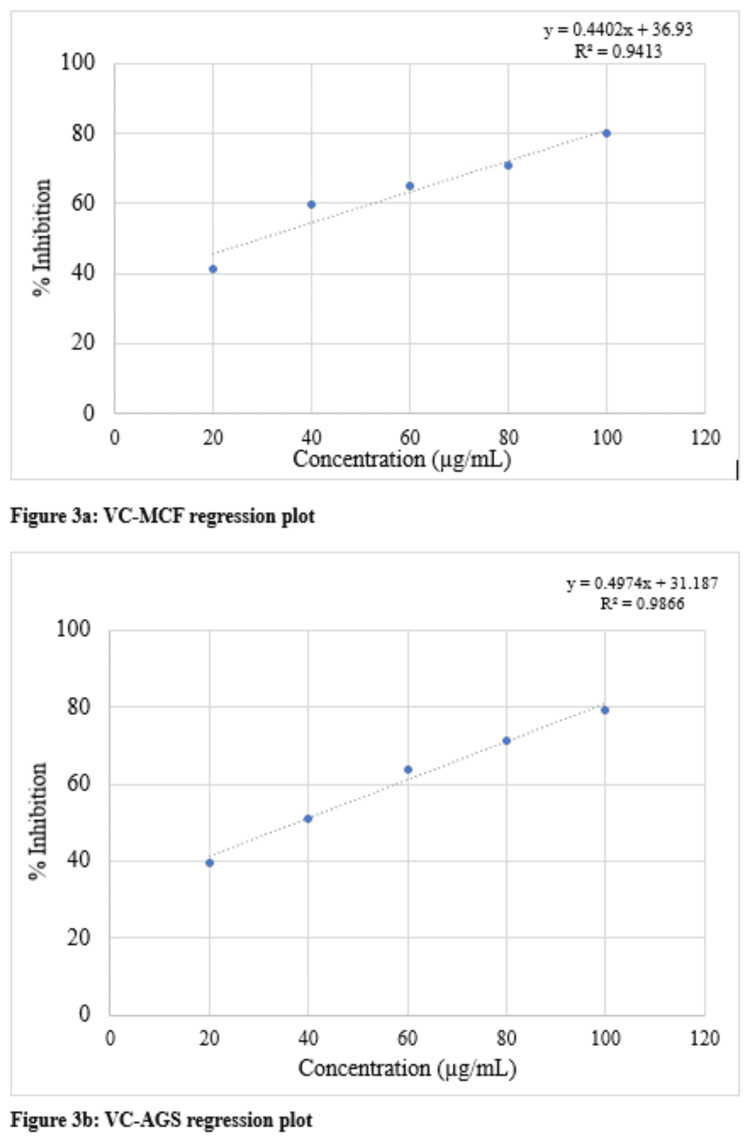
a) Linear regression plot for VC-MCF-7 b) Linear regression plot for VC-AGS. VC: Vincristine, MCF-7: Human mammary gland breast adenocarcinoma cell line, AGS: Human gastric adenocarcinoma cell line, µg/mL: Microgram per milliliter, %: Percentage

Figure 3 emphasizes the inhibitory effect of VC on human cancer cell lines in a dose-dependent manner. Linear regression analysis revealed a robust positive correlation between the percent inhibition and the increasing VC concentrations in both MCF-7 and AGS cell lines. For MCF-7, the regression equation was 𝑦 = 0.4402 𝑥 + 36.93 with 𝑅² = 0.9413, whereas AGS cells exhibited an even stronger fit with 𝑦 = 0.4974 𝑥 + 31.187 and 𝑅² = 0.9866. These results verify the potent cytotoxic activity of VC, as AGS cells exhibit a comparatively higher sensitivity to treatment. 

**Figure 4:**
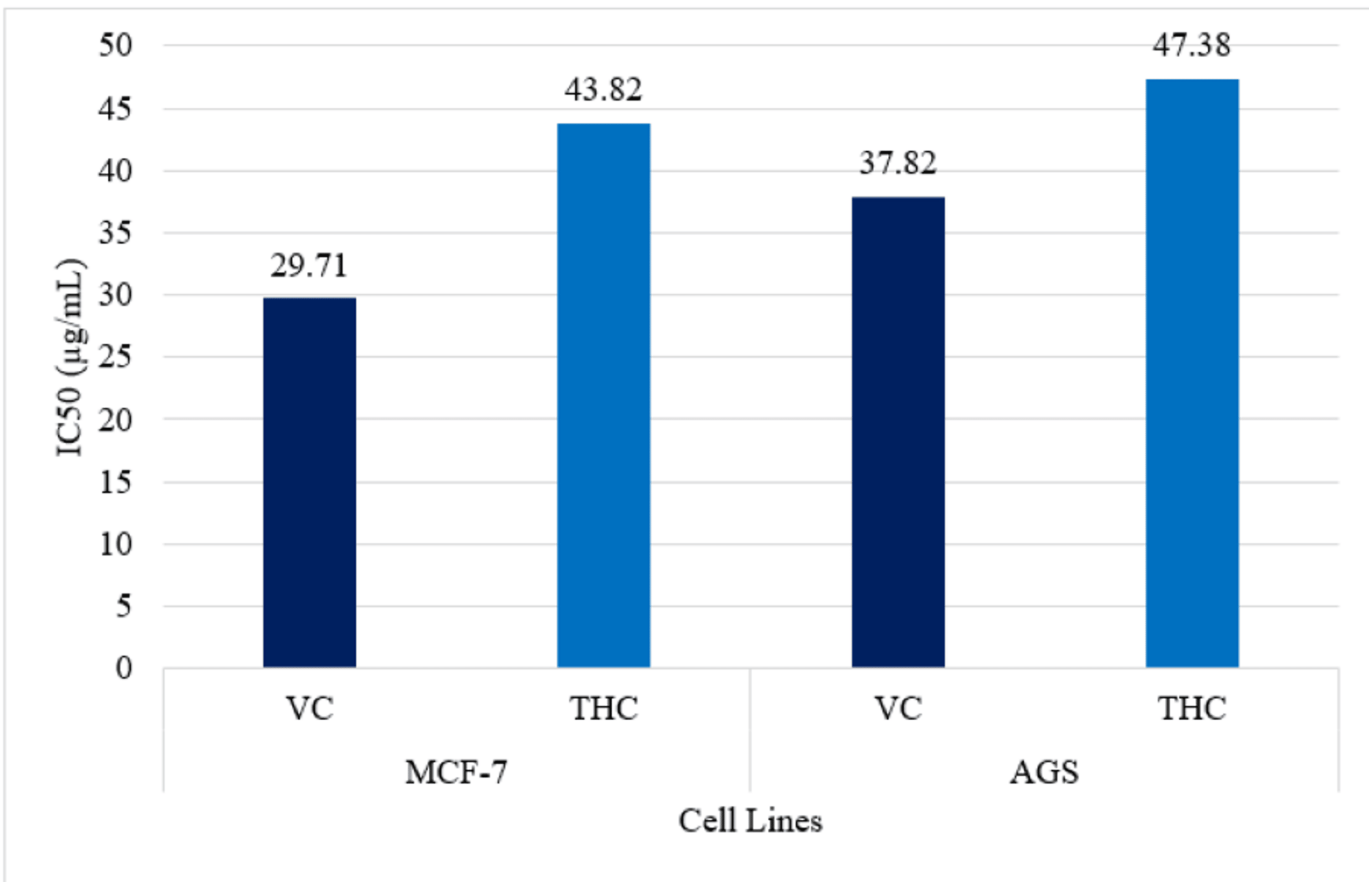
Graphical representation comparing IC50 values for MCF-7 and AGS cell lines against VC and THC. VC: Vincristine, THC: Thiocolchicoside, IC50: Half maximal inhibitory concentration, µg/mL: Microgram per milliliter, MCF-7: Human mammary gland breast adenocarcinoma cell line, AGS: Human gastric adenocarcinoma cell line

The disparity in potency between THC and VC in both cell lines is clearly demonstrated by the graphical display of IC₅₀ values. The consistently lower IC₅₀ values of VC reinforce its position as a more effective antiproliferative agent compared to THC (Figure 4). 

Additional evidence of the concentration-dependent effect of THC and VC is provided by percentage inhibition data. In MCF-7 cells (Table 3), VC demonstrated superior inhibition at all concentrations tested in comparison to THC. Similarly, in AGS cells (Table 4), VC exhibited a more potent inhibitory effect, while THC exhibited moderate activity, with inhibition values increasing consistently from 20 to 100 µg/mL. 

**Table 3:** Percentage inhibition of VC and THC on MCF-7 Cell Lines at different concentrations. THC: Thiocolchicoside, VC: Vincristine, DMSO: Dimethyl sulfoxide, MCF-7: Human mammary gland breast adenocarcinoma cell line, %: Percentage, µg/mL: Micrograms per milliliter

Concentration (µg/mL)	VC(% Inhibition)	THC (% Inhibition)
Control (DMSO)- 200	0	0
20	41.49	35.20
40	59.58	52.91
60	64.84	61.77
80	71.00	62.85
100	79.80	67.05

**Table 4:** Percentage inhibition of VC and THC on AGS cell line at different concentrations. VC: Vincristine, THC: Thiocolchicoside, DMSO: Dimethyl sulfoxide, µg/mL: Microgram per milliliter, AGS: Human gastric adenocarcinoma cell line, %: Percentage

Concentration (µg/mL)	VC(% Inhibition)	THC (% Inhibition)
Control (DMSO) 200	0	0
20	39.61	30.48
40	51.10	46.65
60	63.88	62.53
80	71.31	70.92
100	79.24	75.37

**Figure 5:**
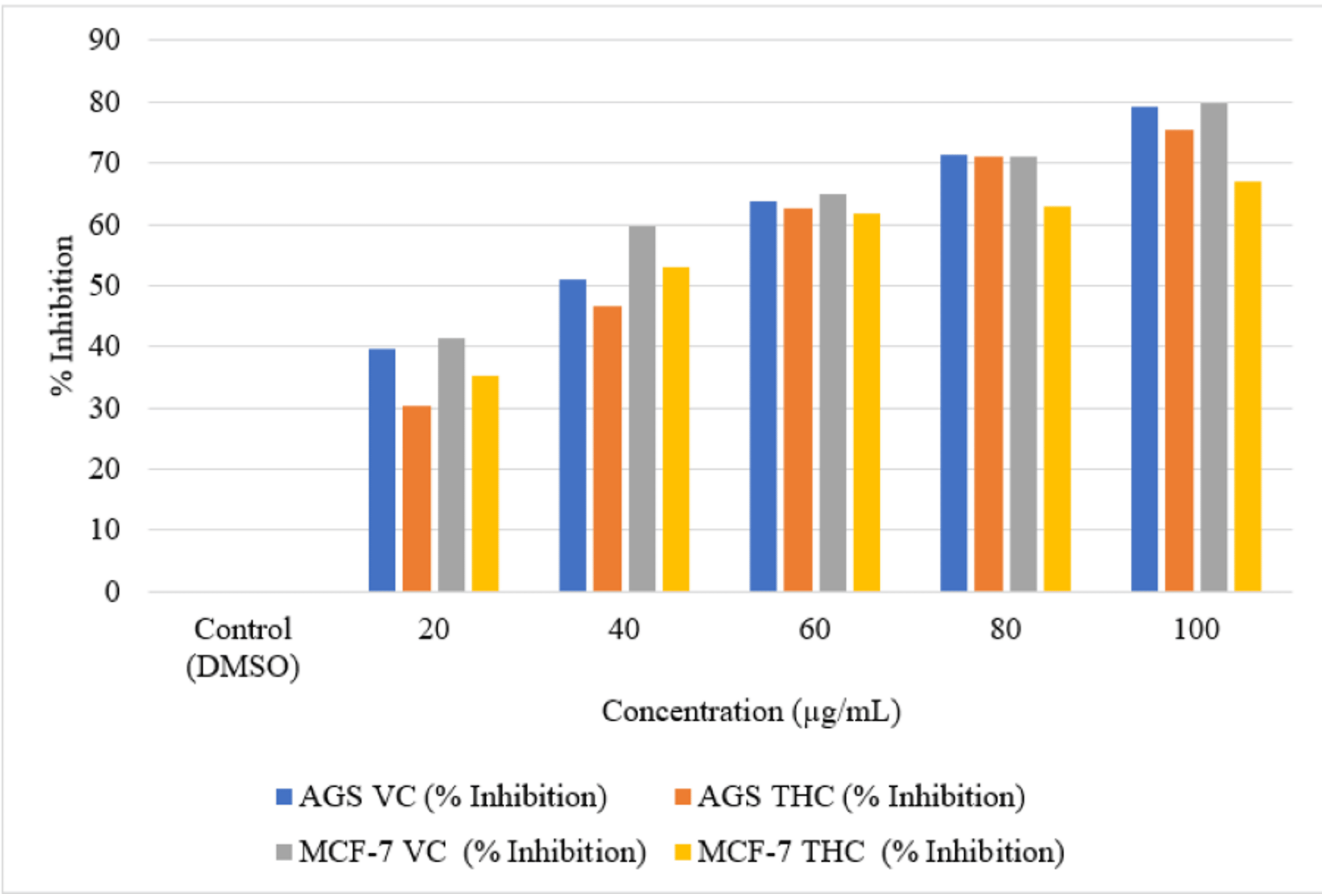
Graphical representation comparing percentage inhibition of VC and THC for antiproliferative activity against MCF-7 and AGS cell lines. VC: Vincristine, THC: Thiocolchicoside, DMSO: Dimethyl sulfoxide, µg/mL: Microgram per milliliter, AGS: Human gastric adenocarcinoma cell line, MCF-7: Human mammary gland breast adenocarcinoma cell line, IC50: Half maximal inhibitory concentration, %: Percentage

The percentage inhibition of VC and THC for antiproliferative activity against MCF-7 and AGS cell lines is compared in a graphical representation (Figure 5). THC exhibits moderate activity that is concentration-dependent, while VC consistently exhibits stronger inhibition. 

## Discussion

The current in vitro study validates that THC demonstrates quantifiable cytotoxic effects on MCF-7 and AGS cancer cells. This activity may be mechanistically linked to its structural resemblance to colchicine, which is recognized for its ability to interfere with microtubule formation and trigger apoptosis [6-9,18]. Prior studies have emphasized the capacity of THC to regulate p53 activation and obstruct NF-κB signaling pathways, supporting our results [11-13]. VC is still potent, but the anticancer efficacy of THC in this study suggests that it could be used as an additional anticancer drug [19]. Drug repurposing has become a significant technique in oncology, presenting the opportunity to discover novel anticancer uses for medicines already sanctioned for different indications [3]. Recent systematic reviews have emphasized the advancements and obstacles of drug repurposing in oncology, underscoring its position as a cost-effective approach for discovering novel anticancer applications of current medications [20]. The idea of drug repositioning was first put forward as a systematic way to find new therapeutic uses for drugs that are already on the market, which is the basis for modern repurposing methods [21]. This method cuts down on development time, cost, and safety worries compared to starting from scratch with a new drug. THC, which has been used as a muscle relaxant [4], is a good candidate for repurposing because it has a structure similar to colchicine and has been shown to be toxic to cancer cells [6,9,22].

**Figure 6:**
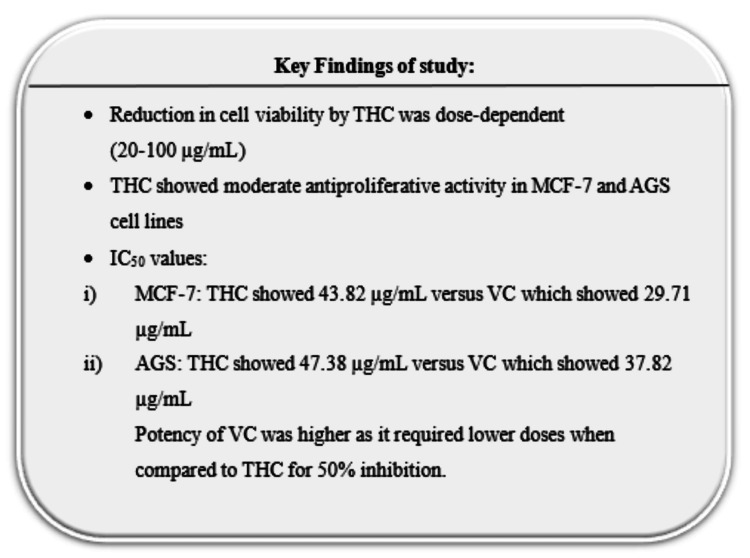
Key findings of the study. THC: Thiocolchicoside, VC: Vincristine, IC50: Half Maximal Inhibitory Concentration, µg/mL: Micrograms per liter

Our discovery of dose-dependent cytotoxicity in MCF-7 and AGS cells corroborates the concept that THC may exhibit anticancer effects via processes involving microtubule breakdown and apoptosis induction [10-13]. Repurposing THC may extend its use beyond neuromuscular disorders, potentially as an adjuvant in cancer therapy. Thalidomide and metformin are two examples of repurposed medications that have worked well in the past. This shows how promising this approach is in current cancer treatment [14,15,23,24]. More research on microtubule-targeting therapies like paclitaxel shows how important this mechanism is in cancer treatment [3,25]. Natural products and phytochemicals have been investigated for anticancer drug development, underscoring the significance of alternate sources of cytotoxic agents [14,15,23]. The study indicated that THC diminished cell viability in a dose-dependent form within the investigated range (20-100 µg/mL). THC had moderate antiproliferative effects in both MCF-7 and AGS cell lines. The determined IC₅₀ values were 43.82 µg/mL for MCF‑7 and 47.38 µg/mL for AGS, signifying observable cytotoxicity. Vincristine (VC) exhibited superior potency, evidenced by lower IC₅₀ values of 29.71 µg/mL for MCF‑7 and 37.82 µg/mL for AGS. The data indicate that THC has a concentration-dependent inhibitory impact, but VC necessitates lower dosages to attain comparable growth suppression, emphasizing its much greater cytotoxic potential (Figure 6).

Limitations

This study is restricted by the absence of mechanistic assays and an in vivo design. Although the MTT assay offered valuable insights into antiproliferative activity, complementary methods, including in vivo validation, cell cycle studies, and apoptosis marker analysis, are required to verify translational relevance. The study was also limited to two cancer cell lines and relied on a single assay method, which may not have captured the complete spectrum of anticancer mechanisms. Additionally, the comparison was limited to a single standard drug, VC; the incorporation of additional reference drugs could have offered a more comprehensive benchmark.

Strengths

Despite these limitations, the study has several strengths. The relative potency of THC against a well-established anticancer agent is underscored by a direct comparison with VC. Quantitative IC₅₀ determination enabled the establishment of distinct concentration-dependent trends for both AGS and MCF-7 cell lines, and the use of multiple dose levels (20-100 µg/mL) provided measurable endpoints. The reliability of the findings was guaranteed by the detailed methodology and triplicate experiments, which improved reproducibility. The study's most significant contribution is the addition of new data on THC's antiproliferative activity, which expands the limited literature and establishes a foundation for future mechanistic and in vivo investigations.

Future research recommendation

In order to ascertain whether the effects of THC are tissue-specific or broadly applicable, future research should broaden the assessment of its anticancer potential across a broader range of cancer cell lines. Mechanistic investigations are necessary to elucidate the molecular processes involved, namely the function of NF‑κB suppression, apoptosis induction, and microtubule interactions. In vivo validation of animal models is crucial for evaluating pharmacokinetics, bioavailability, and toxicity, thereby connecting in vitro results with clinical applicability. Furthermore, investigating combination techniques with existing chemotherapeutics may uncover synergistic effects that improve efficacy or decrease necessary dosages. Innovations in drug delivery systems, particularly nanoparticle-based formulations, may enhance efficacy and reduce off-target effects [26]. Ultimately, meticulously planned clinical trials will be essential to determine the therapeutic role of THC in oncology and to assess its safety and efficacy relative to current treatments. Translation into human studies is essential, as prior research has indicated the efficacy of THC nanogel on KB‑1 cell lines, implying its potential for therapeutic application [26]. Subsequently, a forthcoming study ought to emphasize clinical studies to validate therapeutic significance in cancer patients.

## Conclusions

THC demonstrated dose-dependent cytotoxicity towards MCF-7 and AGS cell lines, albeit with diminished potency relative to VC. These findings offer preliminary evidence that THC, a substance commonly utilized as a muscle relaxant, may have anticancer effects via processes related to microtubule disruption and the triggering of death. This underscores the promise of drug repurposing in oncology, allowing established drugs to be redirected for innovative therapeutic applications. This study, although intriguing, has drawbacks, such as its in vitro methodology and the lack of mechanistic testing. Subsequent research should concentrate on the analysis of apoptotic markers, evaluation of the cell cycle, and in vivo validation to determine translational relevance. Improvements in drug delivery systems, particularly nanoparticle-based formulations, may increase efficacy and diminish off-target effects. Ultimately, well‑designed clinical trials are needed to assess THC’s safety and efficacy, which may expand its use beyond neuromuscular disorders and provide cost‑effective cancer treatment options.
